# Antibiotic association in patients presented to ED with diarrhoea found positive for C. difficile infection-A retrospective study

**DOI:** 10.1186/1865-1380-8-S1-P7

**Published:** 2015-04-22

**Authors:** Rohan Shah, Sagar Shah, Rahul Shah, Sanjay Mehta, Sameer Rathi

**Affiliations:** 1 Kokilaben Dhirubhai Ambani Hospital, Mumbai, India

## Objectives

Diarrhea is common in patients coming to Emergency Department (ED), especially among those who receive antibiotics. Clostridium difficile is the most commonly identified organism for antibiotic associated diarrhea. Because of the frequent use of broad-spectrum antibiotics, the incidence of C. difficile- associated diarrhea (CDAD) has risen dramatically in recent years. Antibiotic exposure has become a recognized risk factor. Data from India are limited to a few studies regarding antibiotic exposure and C. Difficile infection. This retrospective study was done to study the antibiotic association with C. Difficile infection in sick patients who presented to our ED with diarrhea.

## Method

A total of 59 patients with diarrhea were included in this study. They were admitted to our hospital through the ED from January 2013 to June 2014 and whose fecal specimens tested positive for C. difficile toxin. The hospital records of the corresponding patients were retrieved and clinical data were noted. Data including age, sex, duration of diarrhea, clinical features, associated illnesses, exposure to antibiotics, sigmoidoscopic or colonoscopic findings were collected. Data in patients with C. difficile infection (study group) were compared with those in patients without (control group). Testing for toxin A and toxin B of C. difficile was performed on the stool samples using an enzyme-linked immunosorbent assay.

## Results

C. Difficile infections were found to be high in patients who were exposed to cefoperazone+sulbactam (20%), meropenem (19%), piperacillin+tazobactam (15%), ceftriaxone (15%), cefuroxime (12%), colistin (10%) before coming to ED. Other pre-arrival antibiotics exposure associated with C. Difficile infection were for teicoplanin, ciprofloxacin, azithromycin, amoxicillin+clavulanate , tigecycline, imipenem, cefepime, doxycycllin, ertapenem, linezolid, amikacin.

## Conclusion

C. difficile infection rates were high for patients who used cefoperazone+sulbactam, piperacillin+tazobactam, cefuroxime, ceftriaxone, meropenem, colistin before coming to ED. Hence, unnecessary usage of these antibiotics should be avoided and should be limited to critically ill patients and, if they are used, their usage should be justified.

